# P-1656. Incidence, characteristics, and risk factors of ventilator-associated pneumonia in patients with COVID-19

**DOI:** 10.1093/ofid/ofaf695.1831

**Published:** 2026-01-11

**Authors:** Sebastian Ruiz, Alicia Hidron, Ricardo R Díaz, Carlos Agudelo, Gisela De la Rosa, Gustavo Roncancio, Cristian Garcia, Carlos Restrepo, Sara Escobar

**Affiliations:** Hospital San Vicente Fundacion Rionegro, Rionegro, Antioquia, Colombia; Universidad Pontificia Bolivariana; Servicios de Clínica Medihelp, Cartagena, Bolivar, Colombia; SURA EPS, Medellin, Antioquia, Colombia; Hospital Pablo Tobon Uribe, Medellin, Antioquia, Colombia; Clinica CardioVID, Medellín, Antioquia, Colombia; Clínica Universitaria Bolivariana, Medellín, Antioquia, Colombia; Hospital Alma Mater de Antioquia, Medellín, Antioquia, Colombia; EPS Sura, Rionegro, Antioquia, Colombia

## Abstract

**Background:**

SARS-CoV-2 infections have increased the incidence of ventilator- associated pneumonia (VAP) in critically ill patients compared to other viral pneumonias. This study aims to describe the incidence, causative microorganisms, and risk factors associated with VAP in COVID-19 patients.

**Figure:**
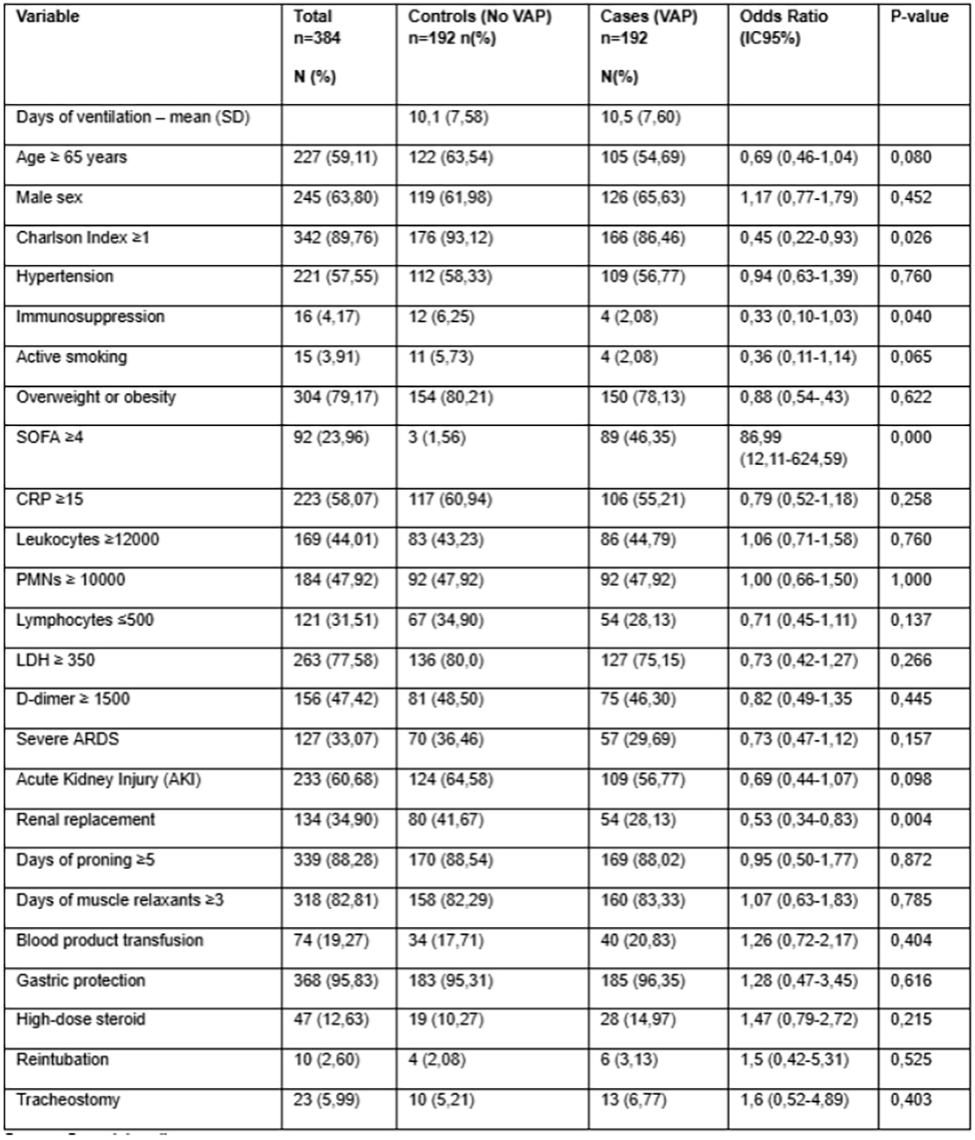
Univariate analysis

**Figure:**
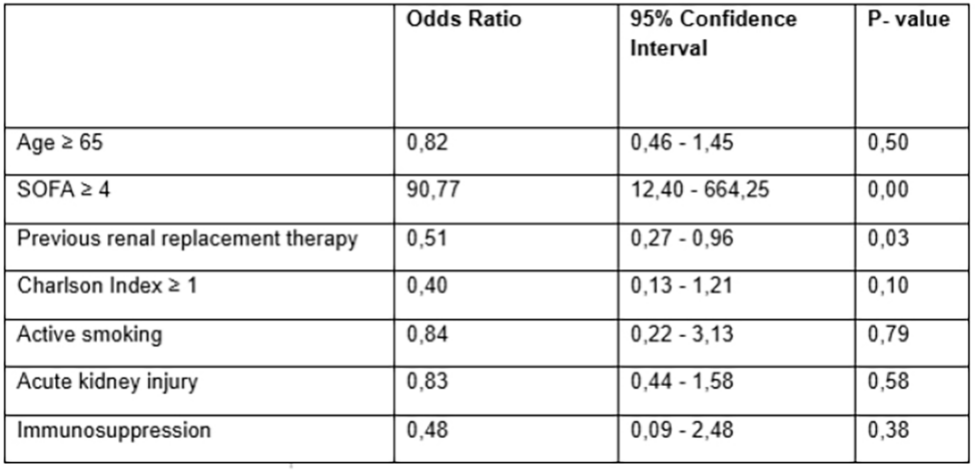
Multivariate analysis

**Methods:**

A retrospective cohort study with a nested case-control design was conducted across five ICUs in Medellín and Rionegro, Colombia, from March to December 2020. Eligible participants included critically ill COVID-19 patients over 18 years old who had been on invasive mechanical ventilation for at least 48 hours. VAP diagnosis followed the Infectious Diseases Society of America and American Thoracic Society (IDSA/ATS) criteria, requiring microbiological, radiological, respiratory, and systemic indicators. Each VAP case was matched to a control by mechanical ventilation duration within ±1 day to account for time-dependent confounding. Data on patient demographics, comorbidities, treatments, and microbiological profiles were collected and analyzed. Conditional logistic regression was used to identify risk factors for VAP.

**Results:**

Among the 449 patients, 196 developed VAP, for a cumulative incidence of 43.65% and an incidence density of 37.3/1,000 ventilator days. The predominant pathogens were Klebsiella pneumoniae (37.3%), Pseudomonas aeruginosa (15.8%), and Staphylococcus aureus (9.4%). A SOFA score of ≥4 was identified as a significant risk factor (OR 90.77, 95% CI 12.40–664.25), while renal replacement therapy was unexpectedly associated with a lower VAP risk (OR 0.51, 95% CI 0.27–0.96).

**Conclusion:**

This study reveals a high VAP incidence among COVID-19 patients, with rates exceeding those reported for non-COVID ICU populations. The predominance of Gram-negative bacteria aligns with global data, though local resistance patterns suggest the need for tailored empirical therapies. The strong association between high SOFA scores and VAP highlights the influence of severe disease on infection risk.

Further research is needed to explore the unexpected association of renal replacement therapy with lower risk. These findings inform targeted VAP prevention and treatment strategies in COVID-19 patients, promoting rational antibiotic use based on local microbiology.

**Disclosures:**

All Authors: No reported disclosures

